# Correction to: Identification hub genes of consensus molecular subtype correlation with immune infiltration and predict prognosis in gastric cancer

**DOI:** 10.1007/s12672-021-00458-x

**Published:** 2021-12-20

**Authors:** Xin Yu, Bin Yu, Weidan Fang, Jianping Xiong, Mei Ma

**Affiliations:** 1 Department of Oncology, The First Afliated Hospital of Nanchang University, Nanchang, 330006 Jiangxi China; 2 Department of General Surgery, The First Afliated Hospital of Nanchang University, Nanchang, 330006 Jiangxi China

## Correction to: Discover Oncology (2021) 12:41 https://doi.org/10.1007/s12672-021-00434-5

Since publication of the original article [1] the authors have become aware that reference 16 [2] has been retracted. This reference has now been replaced with [3]. The original article has been updated.
